# Attendance-Based Adherence and Outcomes of Obesity Management Program in Arab Adolescents

**DOI:** 10.3390/children10091449

**Published:** 2023-08-25

**Authors:** Nasser M. Al-Daghri, Osama E. Amer, Malak N. K. Khattak, Syed D. Hussain, Ghadah Alkhaldi, Hanan A. Alfawaz, Mohamed A. Elsaid, Shaun Sabico

**Affiliations:** 1 Chair for Biomarkers of Chronic Diseases, Biochemistry Department, College of Science, King Saud University, Riyadh 11451, Saudi Arabia; oamer1@ksu.edu.sa (O.E.A.); mkhattak@ksu.edu.sa (M.N.K.K.); shussain@ksu.edu.sa (S.D.H.); malsaeed@ksu.edu.sa (M.A.E.); ssabico@ksu.edu.sa (S.S.); 2 Department of Community Health Sciences, College of Applied Medical Sciences, King Saud University, Riyadh 11451, Saudi Arabia; ghalkhaldi@ksu.edu.sa; 3 Department of Food Science and Nutrition, College of Food Science and Agriculture, King Saud University, Riyadh 11451, Saudi Arabia; halfawaz@ksu.edu.sa

**Keywords:** adherence, educational program, lifestyle, adolescents, obesity

## Abstract

Pediatric obesity has become a global pandemic in the last century, contributing to short and long-term medical conditions that heighten the risk of morbidity and mortality in children. The 12-month school-based obesity management educational program aims to assess the effect of adherence to the lifestyle educational program and target outcomes, obesity, and hypertension. A total of 363 (nonadherent, *N* = 179; adherent, *N* = 184) Saudi school adolescents aged 12–18 were recruited from 60 schools in Riyadh City, Saudi Arabia. Anthropometrics, lipid profile, and blood glucose were measured at baseline and post-intervention. The level of adherence was based on the number of attended educational sessions, and participants were grouped accordingly into two groups: adherent group (attended ≥ 3 sessions) and nonadherent group (attended 1–2 sessions) out of a total of five sessions. Results demonstrated that significantly more participants in the adherent group achieved the primary program goal of reducing obesity indices [body weight, body mass index (BMI), and BMI z-score] than the nonadherent group. Additionally, among adherent obese participants, BMI z-score significantly decreased after the 12-month intervention (post-intervention: 1.5 ± 0.7 vs. baseline: 1.7 ± 0.6, *p* < 0.05), while the trend in BMI z-score modestly increased in the nonadherent obese participants post-intervention (post-intervention: 1.8 ± 0.7 vs. baseline: 1.7 ± 0.6, *p* > 0.05). Moreover, there was a substantial reduction in hypertension prevalence only in the adherent group (*p* = 0.003) and among adherent obese participants in particular (*p* = 0.03). Furthermore, adherence to session attendance was higher in girls than boys, which led to better outcomes among girls than boys. For the secondary outcomes, lipid profile indices increased in both groups, while no changes were observed in the glycemic profile. In conclusion, greater adherence to educational sessions achieved modest but favorable weight changes and improved blood pressure among obese adolescents. Future intervention studies should take into consideration the need to improve attendance to enhance adherence to the program among adolescents at risk.

## 1. Introduction

Obesity is a key risk factor for several chronic, non-communicable diseases, estimated as the principal cause of 74% of all-cause mortality globally as of 2022 [1]. It has been established as a significant risk factor for atherosclerotic disease [2,3] and type 2 diabetes mellitus [4], leading to amplified risk of morbidity and mortality [5]. Consequently, pediatric obesity is increasingly becoming a public health threat affecting most industrialized countries worldwide, influenced by health behaviors, psychosocial factors, biology, and genetics [6]. The key modifiable contributors to childhood obesity are physical inactivity and overnutrition, reflected as visceral adiposity, which, if untreated over time, increases the risk for more serious chronic diseases and mortality, subsequently aggravating the family and community’s economic health burden [7,8]. Compared to individuals who maintained a healthy weight during childhood, obese children and adolescents are five times likelier to remain obese in adulthood [9]. Childhood overweight and obesity prevalence have risen significantly from 4% in 1975 to 18% in 2016 among those aged 5 to 19 years [10]. Obese children and adolescents are more prone to dyslipidemia [2], cardiovascular diseases [11], non-alcoholic fatty liver disease [12], and asthma and lung functioning [13]. Pediatric hypertension is an important healthcare concern. Several epidemiological studies have found that pediatric hypertension prevalence ranges from 3% in the general population to 25% in obese children [14]. Previous research has indicated that pediatric hypertension is linked to obesity and unhealthy behaviors, e.g., increased caloric/salt intake and a sedentary lifestyle [14,15].

In Saudi Arabia, one out of every four girls and one of every three boys aged 12–18 years is overweight or obese [16]. Several lifestyle factors appear to be key drivers for being overweight or obese, including physical inactivity, skipping breakfast, dining outside, and eating too much-sweetened beverages [10]. Locally, there has been a shift from diets composed of whole foods and low in refined oils and sugars to an energy-dense and nutrient-poor diet composed of fat and sugar-rich diets and processed foods [17,18]. Previous intervention studies targeting children and adolescents demonstrated that school-based intervention has the potential to be effective in preventing obesity, as most children and adolescents consume a significant portion of their daily calories and spend half of their waking hours at school [18].

Lifestyle intervention is the primary treatment for childhood obesity as recommended by the National Institute for Health and Care Excellence (NICE) guidance [19]. This includes changes in dietary habits to reduce daily caloric intake [20]. Ojeda-Rodriguez et al. [12] demonstrated that lifestyle intervention (nutritional education, exercise, and reducing calorie intake) successfully reduced BMI standard deviation score in obese children [21]. Recent studies have documented a favorable association between adherence to lifestyle intervention programs, such as compliance with self-monitoring of physical activity and food intake, session attendance and outcomes of the intervention program. Higher levels of session attendance in children have been linked to more substantial weight loss [22,23].

Similarly, it has been shown that successful weight management in adolescents with obesity was related to accurate self-monitoring in lifestyle modification programs [24,25]. Furthermore, evidence pointed out that weight loss in adolescents was associated with parent self-monitoring [26]. Due to the necessity to improve the effectiveness of intervention programs to prevent pediatric obesity, there is a strong rationale to examine the role of adherence in lifestyle intervention programs more closely.

Despite the plethora of successful interventions to prevent and manage childhood obesity [27,28], most lifestyle modification programs done in Saudi Arabia focused on adult obesity and individuals at high risk for diabetes [29,30], while pediatric obesity studies are mostly observational [31,32]. The current study investigates the efficacy of attendance-based adherence to a lifestyle obesity management program and its association with changes in obesity indices and hypertension in a cohort of Arab children and adolescents. The current study hypothesizes that attendance-based adherence is positively associated with the intervention outcomes.

## 2. Materials and Methods

Our study’s primary outcomes are changes in anthropometric indices (body weight, waist, and BMI) and blood pressure. Secondary outcomes are improvements in serum lipids and glycated hemoglobin (HbA1c).

### 2.1. Participants

**Figure 1:**
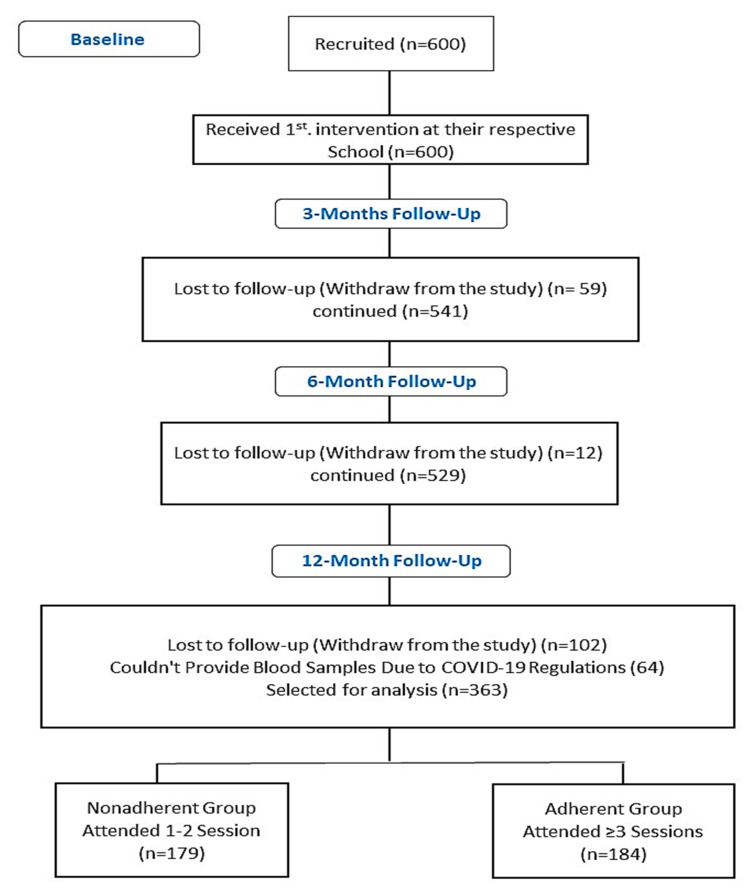
Flow chart of the study participants describing their participation and allocation.

This 12-month (November 2019–November 2020) lifestyle intervention study was a collaborative project by the Chair for Biomarkers of Chronic Diseases (CBCD) at King Saud University (KSU) and the Saudi Charity Association of Diabetes (SCAD). Six hundred (600) healthy school-attending Saudi boys and girls aged 12–18 years from randomly selected 60 preparatory and high schools in Riyadh City, Saudi Arabia. Students with chronic diseases or those on medications that may affect parameters of interest were excluded. Parental informed consent was obtained from each participant before the study started. At baseline, all participants were asked to provide fasting blood samples. Baseline anthropometric and glycemic status were assessed. At the end of the study, due to the COVID-19 lockdown, only 363 participants out of 600 students were able to come for the extraction of fasting blood samples and endpoint measurements. A flow chart describing the study population is provided in Figure 1. Baseline characteristics of participants are provided in Supplementary Table S1. This study was approved by the Institutional Review Board (IRB) of the College of Medicine, King Saud University (KSU), Riyadh, Saudi Arabia (No. E-19-4239, 29 October 2019). An English-translated copy of the brochures provided to the parents is provided as Supplementary File S1.

### 2.2. Intervention

The full protocol, including the materials provided to the participants, was published previously [33,34]. In brief, education on diet and exercise was given to all participants by certified health professionals, with the primary aim of reducing body weight by 5% or more. The topics covered include mindful eating, such as decreasing portion sizes to reduce caloric intake, having low-fat lunches to school and discontinuing juice/soda intake, and increasing physical activity (outdoor if no restrictions) and 30-min aerobic exercise 5 times per week. The exercise type, duration, and frequency were personalized according to their lifestyle or health conditions.

Educational materials were supplied to all study staff to provide consistent educational programs across sites. Also, all participants were given booklets, pamphlets, gamification, and infographic videos about the recommended lifestyle changes. During the 12-month intervention, participants were educated every three months about the essential lifestyle modifications to prevent obesity, the first of five sessions that were given in classrooms. Unfortunately, due to COVID-19 lockdown regulations, the follow-up educational activities were done through a virtual meeting platform (Zoom) and social media platforms: Telegram, WhatsApp, Twitter, and Facebook. Participants’ online attendance (a measure of adherence) was taken using Google Forms during the educational sessions. Overweight and obese participants, in particular, were monitored by phone by a registered nutritionist.

### 2.3. Anthropometrics and Biochemical Parameters

Blood samples and anthropometrics were collected at two time points: baseline and after a 12-month follow-up. Height (cm), weight (cm), body mass index BMI (kg/m^2^), waist (cm) and hip (cm) circumferences, Systolic and diastolic blood pressures (SBP and DBP, respectively, measured as the average of 2 readings with a 15-min interval, using pediatric cut-offs appropriate for children’s sizes). 

All participants were instructed to come in a 10-h overnight fasting state to their respective schools. Fasting blood samples were collected by trained nurses at baseline and after intervention. Fasting blood glucose, high-density lipoprotein cholesterol (HDL-c), total cholesterol (TC), and triglycerides (TG) were measured using a standard routine laboratory analysis (Konelab, Finland). Low-density lipoprotein cholesterol (LDL-c) was calculated using the Friedwald equation. HbA1c was analyzed using the D-10 Hemoglobin Testing System (ion-exchange chromatography) (Bio-Rad, Hercules, CA, USA). Overweight and obese were classified according to sex, age and BMI percentile for children as done previously [35,36]. 

### 2.4. Obesity and Hypertension Cut-Offs

Overweight and obese children and adolescents were defined according to the criteria of the International Obesity Task Force (IOTF) cut-offs [37]. Definition of hypertension, systolic blood pressure (SBP) and/or diastolic blood pressure (DBP) ≥ 95th percentile for age, sex and height based on 2017 clinical practice guidelines [38].

### 2.5. Statistical Analysis

SPSS version 28.0 (SPSS, Inc., Chicago, IL, USA) was used to analyze the data. We used the Kolmogorov-Smirnov test to ensure that our data were normally distributed. Normally distributed data were presented as mean and standard deviation (SD). Non-normal data were presented as median (1st and 3rd quartiles). Non-Gaussian variables were log-transformed before parametric analysis. Categorical variables were shown as frequencies (percentages) and analyzed by the MC-Nemar test. Student independent *t*-test was performed for mean difference between Adherent vs. Non-Adherent at baseline Further Pair Student T-Test was performed to check overtime interval there was mean change in adherent and non-adherent group. General repeated measurement analysis was performed to check the overweight and obese participants’ time interval change effect, time effect by gender and gender, group effect and time effect interval. Statistical significance *p* < 0.05 is considered significant.

The sample size was calculated based on the results reported by Berkowitz et al. [39]. who noticed attendance was significantly associated with achieving a ≥5% reduction in BMI at month 12. Berkowitz et al. revealed that among the participants of a group lifestyle modification program (Group LMP), only 8% of low attendees lost ≥ 5% of initial BMI at month 12, compared with 27% of high attenders (*p* = 0.045). Based on these statistics, the total required sample size at 95% power and 5% level of significance is 182.

## 3. Results

After a 12-month follow-up interrupted by COVID-19 pandemic lockdown regulations, only a total of 363 out of 600 participants (60%) were able to provide follow-up anthropometrics and fasting blood samples. Participants’ online attendance was taken using Google Forms during the educational sessions. Based on the number of attended sessions for each participant, participants were grouped into two groups (nonadherent group, defined as participants who attended 1–2 sessions; and adherent group, defined as participants who attended ≥ 3 sessions). Baseline characteristics of all participants are provided in Supplementary Table S1, as well as differences in the effects of intervention in boys and girls (Supplementary Table S2).

### 3.1. Comparison of Clinical Characteristics in Adherent and Nonadherent Groups after 12-Month Follow-Up

Table 1 shows the pre-and post-intervention clinical characteristics of all participants in both groups. Participants’ weight significantly increased in both groups overtime (*p* < 0.001). BMI significantly decreased in both groups but more in the adherent group (*p* < 0.01). BMI z-score decreased modestly in the adherent group (*p* = 0.09), while it was significantly increased in the nonadherent group (0.093 ± 1.05 vs. −0.015 ± 0.98, *p* < 0.007). The waist was significantly increased only in the nonadherent group (85.6 ± 10.4 vs. 75.0 ± 17.3, *p* < 0.001). Hip circumference significantly increased in the nonadherent group (92.3 ± 11.6 vs. 81.6 ± 23.6, *p* < 0.001), while it significantly decreased in the adherent group (80.6 ± 15.0 vs. 88.8 ± 16.1, *p* < 0.001). WHR significantly increased in both groups (both *p* < 0.001). SBP significantly decreased in both groups over time (nonadherent, 116.6 ± 8.7 vs. 120.6 ± 13.5, *p* = 0.003; adherent, 106.1 ± 13.6 vs. 121.2 ± 16.4, *p* < 0.01). DBP significantly increased over time only in the nonadherent group (75.7 ± 7.1 vs. 68.7 ± 8.8, *p* < 0.001). Lastly, blood glucose, HbA1c, TC, HDL-c, LDL-c, and TG significantly increased in both groups.

### 3.2. Primary Outcomes

Only 23 (6%) participants were able to achieve the study’s main goal (body weight reduction by ≥5%). More participants in the adherent group achieved this goal than in the nonadherent group, but this was not significant (8% vs. 4%, *p* = 0.11) (not mentioned in the table). Furthermore, a significant reduction in the incidence of hypertension among adherent participants was seen overall (*p* = 0.003), and among adherent obese participants only (*p* = 0.03) (Table 2). These were not observed in the normal and overweight participants regardless of adherence status.

### 3.3. Pre- and Post-Intervention Comparison of Overweigh and Obese Participants in Both Groups

Table 3 shows pre- and post-intervention clinical characteristics of overweight and obese participants in both groups. Participants’ weight significantly increased only in the nonadherent group overtime (*p* < 0.01). BMI significantly decreased in both groups, more so in the adherent group (nonadherent, 28.2 ± 4.9 vs. 29.8 ± 4.4; adherent, 26.6 ± 4.7 vs. 29.3 ± 4.5, *p* < 0.01). BMI z-score significantly decreased only in the adherent group (0.87 ± 0.80 vs. 1.05 ± 0.75, *p* < 0.01). BMI z-score. Waist significantly increased in the nonadherent group (88.7 ± 9.5 vs. 81.3 ± 21.8, *p* < 0.05) while significantly decreased in the adherent group (75.7 ± 15.9 vs. 82.2 ± 16.7, *p* < 0.01). SBP significantly decreased post-intervention in both groups while DBP was significantly increased only in the nonadherent group (*p* < 0.01). Lipid profile indices significantly increased in both groups. No changes over time were observed in blood glucose or HbA1c levels in both groups.

### 3.4. Pre- and Post-Intervention Comparison of Overweigh Participants in Both Groups

Table 4 shows the pre- and post-intervention clinical characteristics of overweight participants in both groups. BMI significantly decreased in both groups (*p* < 0.01). WHR significantly increased in both groups (nonadherent: 0.92 ± 0.04 vs. 0.84 ± 0.07, *p* < 0.05; adherent, 0.91 ± 0.07 vs. 0.83 ± 0.12, *p* < 0.01). SBP significantly in both groups (*p* < 0.01). DBP significantly increased only in the nonadherent group (75.4 ± 8.1 vs. 69.3 ± 10.5, *p* < 0.05). Lipid and glycemic profile findings were similar to Table 3. 

### 3.5. Pre- and Post-Intervention Comparison of Obese Participants in Both Groups

Table 5 shows the characteristics of obese participants over time. BMI significantly decreased in both groups (*p* < 0.01). BMI z-score significantly decreased only in the adherent group (*p* < 0.05). WHR significantly increased in both groups (*p* < 0.01). SBP significantly decreased post-intervention in both groups (*p* < 0.01). DBP significantly increased only in the nonadherent group (*p* < 0.01) while it significantly decreased in the adherent group (*p* < 0.05). The rest were similar to Table 3.

## 4. Discussion

The current school-based study investigated the relationship between attendance-adherence to obesity management lifestyle intervention programs and program outcomes with an emphasis on blood pressure. The present investigation is one of the few to focus on pediatric hypertension and the first in Saudi Arabia. 

Hypertension is a key risk factor for cardiovascular disease and has been increasing among pediatrics [40]. The prevalence of hypertension among children is about 3% in the general population and about 25% among obese children [14]. Martinovic et al. [41], in a school-based study on 3254 children aged 7–13 years, reported that childhood obesity raised the probability of child hypertension by 68% (*p* < 0.001). Our results showed positive changes in blood pressure, particularly in adherent participants. This is in line with a previous study on 85 children (aged 5–8 years) found decreased DBP in the intervention group compared to the control group post-intervention [42]. In Saudi Arabia, pediatric hypertension is a growing problem. A previous large national study screened 16,226 pediatrics; results have revealed that blood pressure was steadily increasing with age [43]. Authors found that SBP was annually increasing with an average of 1.44 mmHg among girls and 1.66 mmHg among boys, while DBP was increasing annually by 0.83 mmHg among boys and 0.77 mmHg among girls [43].

Our results showed that adherence was a contributing factor in achieving the primary intervention goal of reducing participants’ body weight by 5% or more. However, the difference between adherence and nonadherence was insignificant, which could be attributed to the small sample size. Kim J et al. [44], in a 16-week nutritional intervention study, included monthly 30-min nutritional sessions on children (mean age 12.4 ± 2.0 years), found significant differences in body weight reduction between adherent and nonadherent children, where adherent children achieved lower body weight post-intervention comparing to nonadherent children (−0.2 kg vs. 3.3 kg, *p* < 0.001). A novel finding, however, under-highlighted in other investigations was the significant improvement in the prevalence of hypertension in the adherent group compared with the nonadherent group. Moreover, results demonstrated that session attendance was associated with a reduction in BMI, where adherent participants achieved better reductions in BMI than non-adherent participants. In our cohort, adherence to session attendance was not associated with glycemic control and didn’t improve lipid profile. Several previous pediatric obesity trials have used sessions as a program adherence proxy [22,23]. 

In the present study, we targeted childhood obesity and hypertension. Both hypertension and obesity have been increasing among pediatrics. Both can track into adulthood, which increases heart disease prevalence and its related mortality [25]. Obese children are at a significantly increasing risk for CVD as they have higher SBP and DBP [26]. Previous research has demonstrated that BMI influences BP in pediatrics [25]. In our study, we have incorporated education about switching to a healthy lifestyle, which included dietary and physical activity changes, which were effective in BMI and BP reductions among our adherent group. Several intervention studies on obese and overweight pediatrics (aged 6–16 years) have demonstrated that weight loss was successful in decreasing BP in pediatrics [25]. These intervention studies (5- to 12-month) have incorporated education, physical activity, and diet and demonstrated that intervention was effective in decreasing SBP [27,28]. In a recent large retrospective study that recruited 101,606 of pediatrics (aged 3–17 years) who were followed for several years, authors found that obese and severely obese participants had increased BP at baseline and greater odds of developing hypertension in adulthood compared with pediatrics with normal BMI [37]. Changes in dietary habits are a significant part of lifestyle modifications in treating obesity-related hypertension [25]. The American Academy of Pediatrics published 2011 an updated dietary recommendation for hypertension treatment [37]. They stated that regardless of the hypertension stage or its etiology, hypertensive children should follow the dietary approaches and lifestyle for cardiovascular health to prevent hypertension [37].

The present results show that about 50% of participants attended at least one session of the intervention sessions, and 50% attended more than 60% of the planned sessions. Our attendance rates were lower than those obtained in studies from the West [22,23]. Kalarchian et al. [22], over a 6-month period, found that 63% of attendance rate and attendance rate was related to weight reduction. Another study by Berkowitz et al. [39] reported a median of 75% attendance rate at intervention sessions. Children with better attendance also had significantly higher BMI reductions than children with less attendance.

In line with previous programs for the management of childhood obesity [45,46], our participants’ BMI z-score significantly reduced post-intervention. We analyzed BMI and BMI z-score instead of the absolute change in weight as our participants were still growing in height. In the present analysis, although participants did not have significant weight loss, BMI and BMI z-score were significantly improved as they still grew in height during the intervention. In previous lifestyle modification intervention studies, authors reported a significant reduction in BMI and BMI z-scores among obese children and adolescents in the intervention group compared to the control group [47,48]. BMI z-score is a pediatric and adolescent obesity assessment tool that considers age and sex into account [48]. Previous research has validated BMI z-scores values for pediatric obesity and demonstrated that BMI z-score was a more accurate obesity indicator for children and adolescents [49,50]. The US Preventive Services Task Force proposed a reduction in BMI z-score with more than 0.20–0.25 could be clinically significant [37]. Moreover, a previous intervention study noted a decrease of ≥0.2 SD in BMI z-score, indicating a successful intervention outcome [48]. It can be speculated that without the current lifestyle intervention, our participants would have continued to gain weight and increased their BMI and BMI z-score.

The present study demonstrates sex differences in program determinants and outcomes, where adherence to session attendance was higher in girls than in boys, with better improvements in program outcomes among girls. Previously, a Canadian study reported that girls were identified to have higher odds of adhering to precautionary COVID-19 measures [51]. To maximize intervention outcomes, reasons for non-adherence among males should be examined in future studies. Gender-adapted strategies to improve compliance have to be developed, for instance, increasing targeted education towards males engaging their families [52]. Additionally, there is a need to reveal sex differences in determinants of low adherence and intervention effects to improve adherence. Sex differences in determinants of adherence have been previously identified [53]. 

An important aspect not investigated in the present study is parental influence. Parental adherence to healthier lifestyles may prove pivotal in controlling childhood weight gain since this approach has been tied to a significantly lower risk of pediatric obesity [54]. Furthermore, the switching from school-based to internet-based implementation of the program was relatively new. Several internet-based studies provided promising outcomes [55,56], but an approach that is personalized and family-based remains superior to other strategies, at least in children [57]. In general, there are still significant gaps in our understanding of how best to tailor interventions that increase children’s adherence, different types of nonadherence (e.g., intentional vs. unintentional), and different preferences for delivery (e.g., technology-mediated vs. face-to-face) [58,59,60].

The present study has some limitations. First, we had no control group. Second, the high dropout rate in our study after intervention limited the actual effect of the intervention in the study duration. The high dropout rate was definitely due to the COVID-19 lockdown and the mobility restrictions during the study period. However, this large dropout might also suggest that sustaining interest in lifestyle change is difficult to do, particularly among our cohort of youth, and they may revert to their previous habits in time. Also, this dropout might indicate difficulties in sustaining interest in lifestyle change, particularly among our study population of children and adolescents, and they may revert with time to their previous unhealthy habits. It is worth noting that a high dropout rate is common in Saudi Arabia for intervention studies. Previously, in a 6-month intervention study on 150 type 2 diabetes Saudi patients, only 61 completed the trial [61]. Another Saudi 18-month lifestyle intervention study started with 180 prediabetes participants, and at the end of the study, only 28 participants completed the study [62]. Behavioral techniques such as self-efficacy, goal-setting and provision of feedback should be applied more aggressively in future studies. Lastly, the study focused on weight loss and hypertension and not on biochemical parameters since glycemic and lipid parameters undergo fluctuations within the normal range during growth, as seen in the present results. Therefore, the increase in glucose and lipid profile observed over time may not necessarily reflect that the participants’ lifestyle got worse after intervention but more of a combination of physiological changes and external factors, some of which were not accounted for in the present study [63,64]. Despite these limitations, this study highlights the modest but positive effects of adherence in lifestyle intervention, particularly in the reduction of pediatric hypertension among obese youth. Apart from obesity management, lifestyle interventions with healthy dietary and exercise behaviors have been effective in achieving favorable metabolic effects [65]. Additionally, lifestyle modification approaches were demonstrated previously to have positive effects that can be long-lasting [66,67].

## 5. Conclusions

Our results indicate that greater participant adherence to the intervention improved target outcomes, particularly in reducing pediatric hypertension among Arab obese adolescents. Our results add to the current literature and have clinical value, given the ethnic and cultural variations in compliance with such an obesity management intervention program. Future intervention studies should be geared towards improving adherence by direct involvement of parents and a more personalized approach involving a multi-disciplinary team, especially among children at the highest risk of obesity-related complications. The use of a virtual platform in the implementation of lifestyle modifications among Saudi children still has a lot of room for improvement, including, but not limited to, the addition of educational content that is both encouraging and entertaining to children, which hopefully can translate to better adherence.

## Figures and Tables

**Table 1:** Comparison of clinical characteristics in both groups after 12-month follow-up.

Parameters	Overall					
	Nonadherent		*p*-Value	Adherent		*p*-Value
	Baseline	Follow-Up		Baseline	Follow-Up	
N	179			184		
Age	14.97 ± 1.6			14.89 ± 1.7		
Weight (Kg)	58.2 ± 18.3	60.6 ± 17.2	<0.001	58.3 ± 17.5	60.2 ± 15.5	<0.001
BMI (kg/m^2^)	22.9 ± 5.9	22.1 ± 5.9	<0.001	23.82 ± 6.1	22.10 ± 5.6	<0.001
BMI Z-score	−0.015 ± 0.98	0.093 ± 1.05	0.007	0.117 ± 1.0	0.069 ± 0.96	0.09
Waist (cm)	75.0 ± 17.3	85.6 ± 10.4	<0.001	74.2 ± 14.6	73.4 ± 15.6	0.29
Hip (cm)	81.6 ± 23.6	92.3 ± 11.6	<0.001	88.8 ± 16.1	80.6 ± 15.0	<0.001
WHR	0.86 ± 0.10	0.92 ± 0.10	<0.001	0.84 ± 0.12	0.91 ± 0.07	<0.001
SBP (mmHg)	120.6 ± 13.5	116.6 ± 8.7	0.003	121.2 ± 16.4	106.1 ± 13.6	<0.001
DBP (mmHg)	68.7 ± 8.8	75.7 ± 7.1	<0.001	72.9 ± 11.6	71.7 ± 6.5	0.12
Glucose (mmol/L)	5.3 ± 1.0	5.6 ± 2.2	0.03	5.2 ± 0.6	5.5 ± 2.3	0.06
HbA1c (%)	5.2 ± 0.6	5.3 ± 1.1	0.04	5.1 ± 0.6	5.3 ± 1.3	0.007
TC (mmol/L)	4.4 ± 0.8	6.0 ± 1.2	<0.001	4.4 ± 0.7	5.8 ± 1.4	<0.001
HDL-c (mmol/L)	1.0 ± 0.2	1.5 ± 0.5	0.001	0.95 ± 0.3	1.5 ± 0.6	<0.001
LDL-c (mmol/L)	2.9 ± 0.7	3.7 ± 1.1	<0.001	3.0 ± 0.6	3.7 ± 1.2	<0.001
TG (mmol/L)	1.1 ± 0.5	1.8 ± 0.8	<0.001	1.1 ± 0.6	1.7 ± 0.8	<0.001

Note: Data Presented mean ± SD. *p*-value significant at 0.05 and 0.01 level for baseline and after follow-up. BMI, Body Mass Index; WHR, waist-hip ratio; SBP, Systolic Blood Pressure; DBP, Diastolic Blood Pressure; HbA1c, glycated hemoglobin; TC, Total Cholesterol; HDL-c, High-Density Lipoprotein Cholesterol; LDL-c, Low-Density Lipoprotein Cholesterol; TG, Triglycerides.

**Table 2:** Prevalence of hypertension pre- and post-intervention among study groups.

Group (Total *N*)	Baseline	Follow-Up	*p*-Value
Overall (*N* = 363)	69 (19)	51 (14)	0.16
Nonadherent (*N* = 179)	21 (12)	31 (17)	0.23
Adherent (*N* = 184)	48 (26)	20 (11)	0.003
Normal body weight (*N* = 223)			
Nonadherent (*N* = 122)	9 (7)	17 (14)	0.15
Adherent (*N* = 101)	23 (23)	14 (14)	0.18
Overweight (*N* = 72)			
Nonadherent (*N* = 28)	6 (21)	7 (25)	0.73
Adherent (*N* = 44)	10 (23)	4 (9)	0.18
Obese (*N* = 68)			
Nonadherent (*N* = 29)	6 (25)	4 (14)	0.73
Adherent (*N* = 39)	15 (38)	5 (13)	0.03

Note: Data presented N (%). *p*-value significant at <0.05 using Mc-Nemar test.

**Table 3:** Comparison of clinical characteristic in overweight and obese participants of both groups after 12-month follow-up.

Parameters	Overall Overweight & Obese		Nonadherent		Adherent		*p* *	*p* **	*p* ***
	Baseline	Follow-Up	Baseline	Follow-Up	Baseline	Follow-Up			
N (M/F)	140 (89/51)		57 (51/6)		83 (38/45)				
Age (years)	15.0 ± 1.7		15.2 ± 1.7		14.9 ± 1.6		0.35		
Weight (kg)	74.5 ± 15.1	74.9 ± 13.8	77.1 ± 15.9	78.4 ± 15.0 **	73.2 ± 14.5	73.1 ± 12.8	0.08	0.28	0.17
BMI (kg/m^2^)	29.5 ± 4.5	27.2 ± 4.8 **	29.8 ± 4.4	28.2 ± 4.9 **	29.3 ± 4.5	26.6 ± 4.7 **	0.35	0.15	0.18
BMI Z-score	1.1 ± 0.7	0.99 ± 0.8 *	1.2 ± 0.7	1.2 ± 0.9	1.05 ± 0.75	0.87 ± 0.80 **	0.22	0.13	0.18
Waist (cm)	81.9 ± 18.6	80.3 ± 15.2	81.3 ± 21.8	88.7 ± 9.5 *	82.2 ± 16.7	75.7 ± 15.9 **	<0.001	<0.001	0.02
Hip (cm)	95.1 ± 21.3	86.9 ± 14.5 **	90.6 ± 25.6	94.6 ± 10.4	97.4 ± 18.4	82.7 ± 14.9 **	0.002	<0.001	0.03
WHR	0.85 ± 0.10	0.92 ± 0.05 **	0.88 ± 0.10	0.93 ± 0.04 **	0.84 ± 0.10	0.92 ± 0.06 **	0.46	0.15	0.95
SBP (mmHg)	125.7 ± 15.4	110.9 ± 12.6 **	125.5 ± 13.0	117.0 ± 8.3 **	125.9 ± 16.6	107.6 ± 13.4 **	0.02	<0.001	0.26
DBP (mmHg)	72.5 ± 11.1	74.2 ± 7.2	69.6 ± 9.9	76.9 ± 7.4 **	74.1 ± 11.5	72.8 ± 6.8	<0.001	<0.001	0.095
Glucose (mmol/L)	5.4 ± 1.1	5.8 ± 2.7	5.6 ± 1.7	5.8 ± 2.1	5.3 ± 0.6	5.9 ± 3.0	0.89	0.71	0.95
HbA1c (%)	5.2 ± 0.7	5.5 ± 1.6	5.3 ± 0.7	5.2 ± 0.9	5.2 ± 0.7	5.6 ± 1.8	0.06	0.03	0.50
TC (mmol/L)	4.6 ± 0.7	6.0 ± 1.4 **	4.6 ± 0.8	6.1 ± 1.2 **	4.6 ± 0.7	5.9 ± 1.5 **	<0.001	0.047	0.46
HDL-c (mmol/L)	0.9 ± 0.3	1.4 ± 0.6 **	1.0 ± 0.2	1.5 ± 0.4 **	0.9 ± 0.3	1.4 ± 0.6 **	0.002	<0.001	0.04
LDL-c (mmol/L)	3.1 ± 0.7	3.8 ± 1.2 **	3.1 ± 0.8	3.8 ± 1.0 **	3.1 ± 0.6	3.8 ± 1.3 **	<0.001	<0.001	0.06
TG (mmol/L)	1.2 ± 0.6	1.7 ± 0.8 **	1.3 ± 0.8	1.9 ± 0.7 **	1.2 ± 0.6	1.7 ± 0.9 **	0.002	0.12	0.84

Note: Data Presented as mean ± SD; * and ** denotes *p*-value significant at 0.05 and 0.01 level pre- and post-intervention; *p* * denotes time effect; *p* ** denotes time effect * Sex; *p* *** denotes Sex * group * Time effect. BMI, Body Mass Index; WHR, Waist-Hip Ratio; SBP, Systolic Blood Pressure; DBP, Diastolic Blood Pressure; HbA1c, glycated hemoglobin; TC, Total Cholesterol; HDL-c, High-Density Lipoprotein Cholesterol; LDL-c, Low-Density Lipoprotein Cholesterol; TG, Triglycerides.

**Table 4:** Comparison of clinical characteristic in overweight participants of both groups after 12-month follow-up.

Parameters	Overall Overweight		Nonadherent		Adherent		*p* *	*p* **	*p* ***
	Baseline	Follow-Up	Baseline	Follow-Up	Baseline	Follow-Up			
N (M/F)	72 (38/34)		28 (22/6)		44 (16/28)				
Age (years)	15.2 ± 1.7		15.3 ± 1.8		15.1 ± 1.6		0.66		
Weight (kg)	64.4 ± 10.3	65.9 ± 9.5 **	66.3 ± 10.3	68.2 ± 9.5 **	63.5 ± 10.3	64.7 ± 9.3 *	0.001	0.04	0.94
BMI (kg/m^2^)	26.0 ± 1.7	24.0 ± 3.2 **	26.4 ± 1.6	24.8 ± 3.3 **	25.8 ± 1.8	23.6 ± 3.1 **	0.35	0.51	0.49
BMI Z-score	0.54 ± 0.30	0.47 ± 0.58	0.63 ± 0.27	0.67 ± 0.605	0.50 ± 0.31	0.40 ± 0.55	0.50	0.43	0.41
Waist (cm)	74.6 ± 17.1	78.1 ± 15.4	69.8 ± 21.3	88.6 ± 9.4	77.2 ± 13.9	72.3 ± 15.0	<0.001	0.02	0.28
Hip (cm)	89.8 ± 20.4	85.1 ± 14.9	82.3 ± 25.6	95.9 ± 11.2 **	93.7 ± 16.1	79.4 ± 13.6 **	<0.001	<0.001	0.007
WHR	0.8 ± 0.11	0.9 ± 0.06 **	0.8 ± 0.07	0.9 ± 0.04 *	0.83 ± 0.12	0.91 ± 0.07 **	0.29	0.47	0.54
SBP (mmHg)	124.0 ± 15.4	109.1 ± 12.5 **	124.4 ± 11.8	116.9 ± 9.2 *	123.8 ± 16.9	105.1 ± 12.1 **	0.18	0.003	0.15
DBP (mmHg)	70.9 ± 10.9	73.8 ± 7.8	69.3 ± 10.5	75.4 ± 8.1 *	71.8 ± 11.2	72.9 ± 7.6	0.003	0.02	0.77
Glucose (mmol/L)	5.4 ± 1.5	5.7 ± 2.4	5.8 ± 2.3	5.7 ± 2.4	5.2 ± 0.7	5.63 ± 2.4	0.52	0.39	0.88
HbA1c (%)	5.2 ± 0.8	5.3 ± 1.3	5.3 ± 0.9	5.2 ± 0.8	5.2 ± 0.7	5.37 ± 1.5	0.001	0.001	0.83
TC (mmol/L)	4.5 ± 0.8	5.8 ± 1.2 **	4.4 ± 0.8	5.7 ± 1.1 **	4.6 ± 0.8	5.86 ± 1.3 **	0.001	0.13	0.32
HDL-c (mmol/L)	0.9 ± 0.3	1.6 ± 0.5 **	1.0 ± 0.2	1.6 ± 0.3 **	0.9 ± 0.3	1.56 ± 0.6 **	0.60	0.003	0.55
LDL-c (mmol/L)	3.1 ± 0.7	3.5 ± 1.1 **	2.9 ± 0.7	3.3 ± 1.04	3.2 ± 0.7	3.62 ± 1.1 *	<0.001	0.001	0.09
TG (mmol/L)	1.2 ± 0.7	1.6 ± 0.7 **	1.2 ± 0.5	1.7 ± 0.7 **	1.2 ± 0.7	1.6 ± 0.7 *	0.22	0.82	0.79

Note: Data Presented as mean ± SD; * and ** denotes *p*-value significant at 0.05 and 0.01 level pre- and post-intervention; *p* * denotes time effect; *p* ** denotes time effect * Sex; *p* *** denotes group * time effect. BMI, Body Mass Index; WHR, Waist-Hip Ratio; SBP, Systolic Blood Pressure; DBP, Diastolic Blood Pressure; HbA1c, glycated hemoglobin; TC, Total Cholesterol; HDL-c, High Density Lipoprotein Cholesterol; LDL-c, Low Density Lipoprotein Cholesterol; TG, Triglycerides.

**Table 5:** Comparison of clinical characteristic in obese participants of both groups after 12-month follow-up.

Parameters	Overall Overweight		Nonadherent		Adherent		*p* *	*p* **	*p* ***
	Baseline	Follow-Up	Baseline	Follow-Up	Baseline	Follow-Up			
N (M/F)	68 (51/17)		29 (29/0)		39 (22/17)				
Age (years)	14.9 ± 1.7		15.1 ± 1.6		14.7 ± 1.7		0.34		
Weight (kg)	85.1 ± 11.6	84.5 ± 10.9	87.5 ± 13.4	88.2 ± 12.6	83.7 ± 10.4	82.3 ± 9.3 *	0.53	0.45	0.16
BMI (kg/m^2^)	33.1 ± 3.4	30.5 ± 3.9 **	33.0 ± 3.7	31.5 ± 3.9 **	33.2 ± 3.3	30.0 ± 3.8 **	0.74	0.06	0.51
BMI Z-score	1.7 ± 0.58	1.6 ± 0.70	1.7 ± 0.63	1.8 ± 0.73	1.7 ± 0.6	1.5 ± 0.7 *	0.13	0.08	0.55
Waist (cm)	89.2 ± 17.2	82.6 ± 14.9 *	92.9 ± 15.6	88.8 ± 9.7	87.2 ± 17.9	79.1 ± 16.2 *	<0.001	0.001	0.62
Hip (cm)	100.6 ± 21.0	88.8 ± 14.1 **	99.0 ± 23.2	93.3 ± 9.7	101.4 ± 19.9	86.2 ± 15.6	0.06	0.008	0.87
WHR	0.9 ± 0.09	0.9 ± 0.04 **	0.9 ± 0.11	1.0 ± 0.02	0.8 ± 0.08	0.9 ± 0.05	0.73	0.47	0.30
SBP (mmHg)	127.6 ± 15.3	112.9 ± 12.6 **	126.6 ± 14.2	117.1 ± 7.5 **	128.2 ± 15.9	110.5 ± 14.3 **	0.002	<0.001	0.43
DBP (mmHg)	74.1 ± 11.2	74.8 ± 6.6	69.9 ± 9.5	78.3 ± 6.4 **	76.6 ± 11.5	72.7 ± 5.9 *	<0.001	0.002	0.06
Glucose (mmol/L)	5.4 ± 0.5	6.0 ± 3.1	5.3 ± 0.5	5.8 ± 1.8	5.4 ± 0.5	6.1 ± 3.6	0.72	0.95	0.90
HbA1c (%)	5.2 ± 0.6	6.6 ± 1.8	5.3 ± 0.6	5.19 ± 0.9	5.22 ± 0.6	5.89 ± 2.1	0.79	0.96	0.23
TC (mmol/L)	4.7 ± 0.7	6.1 ± 1.6 **	4.9 ± 0.8	6.6 ± 1.1 **	4.6 ± 0.6	5.9 ± 1.7 **	0.006	0.37	0.94
HDL-c (mmol/L)	1.0 ± 0.2	1.3 ± 0.6 **	1.0 ± 0.11	1.4 ± 0.5 **	0.9 ± 0.3	1.2 ± 0.6 *	<0.001	<0.001	0.002
LDL-c (mmol/L)	3.2 ± 0.7	4.1 ± 1.3 **	3.2 ± 0.8	4.3 ± 0.7 **	3.1 ± 0.6	4.0 ± 1.5	<0.001	0.02	0.51
TG (mmol/L)	1.3 ± 0.6	1.9 ± 0.9 **	1.4 ± 0.7	2.0 ± 0.6 *	1.2 ± 0.6	1.8 ± 1.10 **	0.002	0.04	0.37

Note: Data Presented as mean ± SD; * and ** denotes *p*-value significant at 0.05 and 0.01 level pre- and post-intervention; *p* * denotes time effect; *p* ** denotes time effect * Sex; *p* *** denotes group * time effect. BMI, Body Mass Index; WHR, Waist Hip Ratio; SBP, Systolic Blood Pressure; DBP, Diastolic Blood Pressure; HbA1c, glycated hemoglobin; TC, Total Cholesterol; HDL-c, High Density Lipoprotein Cholesterol; LDL-c, Low Density Lipoprotein Cholesterol; TG, Triglycerides.

## Data Availability

The data presented in this study are available on request from the corresponding author. The data are not publicly available due to privacy protection.

## Supplementary Materials

The following supporting information can be downloaded at https://www.mdpi.com/article/10.3390/children10091449/s1, File S1: Study brochure distributed to parents and participants; Table S1: Baseline clinical characteristic of all participants; Table S2: Pre- and post-intervention comparison of boys and girls in both groups.
