# Supplementary material for: Attendance-Based Adherence and Outcomes of Obesity Management Program in Arab Adolescents
Source: Children (Basel). 2023 Aug 25;10(9):1449. doi: 10.3390/children10091449 (PMC10529466; doi:10.3390/children10091449)
Supplement: Supplementary file 1 [file children-10-01449-s001.zip › children-2549860-supplementary.pdf]

Table S1: Baseline clinical characteristic of all participants

**Parameters**	**Nonadherent**	**Adherent**	**P-Value**
N	154	209	
M/F	124/30	87/122	
Overweight and obese (%)	32 (35/17)	44 (49/39)	
Hypertension (%)	14	23	
Age (years)	14.9 ± 1.6	14.9 ± 1.7	0.66
Height (cm)	158.7 ± 9.5	156.0 ± 9.4	0.003
Weight (kg)	58.2 ± 18.3	58.3 ± 17.5	0.98
BMI (kg/m^2)	22.9 ± 6.0	23.8 ± 6.1	0.19
BMI Z-score	-0.01 ± 0.99	0.12 ± 1.0	0.26
Waist (cm)	74.6 ± 17.5	74.2 ± 14.6	0.80
Hip (cm)	81.8 ± 23.5	88.7 ± 19.8	0.001
WHR	0.86 ± 0.09	0.84 ± 0.11	0.12
SBP (mmHg)	120.6 ± 13.5	121.2 ± 16.4	0.73
DBP (mmHg)	68.7 ± 8.8	72.8 ± 11.6	<0.001
Glucose (mmol/l)	5.25 ± 1.0	5.23 ± 0.70	0.84
HbA1C (%)	5.20 ± 0.6	5.07 ± 0.56	0.22
TC (mmol/l)	4.33 ± 0.8	4.44 ± 0.71	0.20
HDL-C (mmol/l)	1.01 ± 0.2	0.96 ± 0.26	0.04
LDL-C (mmol/l)	2.83 ± 0.7	2.98 ± 0.6	0.03
TG (mmol/l)	1.09 ± 0.5	1.08 ± 0.56	0.92

Note: Data Presented mean ± SD. *P*-value significant at 0.01 and 0.05 level. BMI, Body Mass Index; WHR, Waist Hip Ratio; SBP, Systolic Blood Pressure; DBP, Diastolic Blood Pressure; HbA1C, glycated hemoglobin; TC, Total Cholesterol; HDL-c, High Density Lipoprotein Cholesterol; LDL-c, Low Density Lipoprotein Cholesterol; TG, Triglycerides.

Table S2: Pre- and post-intervention clinical characteristic of boys and girls

**Parameters**	**Males**				***P***	**Females**				***P***
	**Nonadherent**		**Adherent**			**Nonadherent**		**Adherent**		
	**Baseline**	**Follow-Up**	**Baseline**	**Follow-Up**		**Baseline**	**Follow-Up**	**Baseline**	**Follow-Up**	
N	124		87			30		122		
Age (years)	14.9±1.5		15.1±1.6		0.64	15.1±1.9		14.8±1.7		0.42
Weight (Kg)	59.9±19.6	62.6±18.3**	62.3±20.2	64.4±18.2**	0.46	51.2±8.6	52.8±7.9**	55.5±14.8	57.2±12.6**	0.11
BMI (kg/m2)	23.3±6.4	22.3±6.4**	24.5±6.8	23.1±6.7**	0.29	21.3±3.5	21.4±3.7	23.4±5.6	21.4±4.6**	0.30
BMI Z-score	0.04±1.07	0.11±1.14	0.16±1.10	0.19±1.15	0.57	-0.23±0.59	0.03±0.64**	0.09±0.9	-0.01±0.81*	0.42
Waist (cm)	76.2±18.9	87.6±9.3**	76.5±18.3	88.3±10.8**	0.75	70.8±8.9	78.6±11.4*	72.7±11.2	63.3±8.7**	<0.001
Hip (cm)	78.9±25.3	92.3±11.2**	86.3±20.1	93.1±11.4**	0.047	92.0±8.9	92.1±13.3	90.5±12.5	72.1±10.6**	<0.001
WHR	0.89±0.08	0.94±0.05**	0.89±0.08	0.95±0.03**	0.50	0.76±0.06	0.85±0.16*	0.80±0.13	0.88±0.07**	0.03
SBP (mmHg)	121 ±13.8	117.7±7.6	117.8±15.6	117.9±9.2	0.29	120.7±12.6	111.9±11.2**	123.7±16.6	97.3±9.0**	0.004
DBP (mmHg)	67.3±8.1	75.7±7.5**	67.3±10.1	73.9±8.1**	0.30	74.4±9.5	75.6±5.6	77.1±10.9	70.1±4.3**	0.21
Glucose (mmol/l)	5.3±1.1	5.7±2.3	5.2±0.6	5.8±1.7**	0.87	5.0±0.5	5.4±1.8	5.2±0.6	5.3±2.6	0.74
HbA1C (%)	5.2±0.7	5.3±1.04	5.3±0.6	5.2±1.3	0.81	4.9±0.4	5.6±1.2**	4.9±0.5	5.5±1.39**	0.67
TC (mmol/l)	4.4±0.8	6.1±1.1**	4.6±0.7	6.2±1.4**	0.21	4.2±0.8	5.4±1.3**	4.4±0.7	5.6±1.3**	0.25
HDL-C (mmol/l)	1.0±0.2	1.4±0.4**	1.0±0.18	1.1±0.51	<0.001	0.98±0.3	1.8±0.3**	0.90±0.3	1.7±0.6**	0.13
LDL-C (mmol/l)	2.9±0.7	3.9±0.9**	3.0±0.6	4.2±1.2**	0.02	2.9±0.6	3.0±1.1	3.0±0.6	3.3±1.1	0.08
TG (mmol/l)	1.1±0.5	1.9±0.8**	1.2±0.7	2.0±0.9**	0.28	0.94±0.4	1.4±0.6**	1.0±0.4	1.4±0.6**	0.57

Note: Data Presented mean ± SD. * and ** represented p-value significant at 0.05 and 0.01 level for pre- and post-intervention and p-value was obtained for group effect for males and females subjects. BMI, Body Mass Index; WHR, Waist Hip Ratio; SBP, Systolic Blood Pressure; DBP, Diastolic Blood Pressure; HbA1C, glycated hemoglobin; TC, Total Cholesterol; HDL-c, High Density Lipoprotein Cholesterol; LDL-c, Low Density Lipoprotein Cholesterol; TG, Triglycerides.
